# Can facts trump unconditional trust? Evidence-based information halves the influence of physicians’ non-evidence-based cancer screening recommendations

**DOI:** 10.1371/journal.pone.0183024

**Published:** 2017-08-23

**Authors:** Odette Wegwarth, Gert G. Wagner, Gerd Gigerenzer

**Affiliations:** 1 Max Planck Institute for Human Development, Harding Center for Risk Literacy, Berlin, Germany; 2 German Institute for Economic Research and Max Planck Institute for Human Development, Berlin, Germany; Technion Israel Institute of Technology, ISRAEL

## Abstract

Informed decision making in medicine, defined as basing one’s decision on the best current medical evidence, requires both informed physicians *and* informed patients. In cancer screening, however, studies document that these prerequisites are not yet met. Many physicians do not know or understand the medical evidence behind screening tests, do not adequately counsel (asymptomatic) people on screening, and make recommendations that conflict with existing guidelines on informed choice. Consistent with this situation, nation-wide studies showed that the general public misperceives the contribution of cancer screening but that understanding considerably improves when evidence-based information is provided. However, can evidence-based patient information about cancer screening make people also less likely to simply follow a physician’s non-evidence-based advice? A national sample of 897 German citizens, surveyed in face-to-face computer-assisted personal interviews, received either evidence-based (e.g., absolute risks on benefits *and* harms; *n* = 451) or non-evidence-based (e.g., relative risks on benefits only; *n* = 446) patient information about a cancer screening test and were then asked to make their initial cancer screening choice. Thereafter, participants received a hypothetical physician’s recommendation, which was non-evidence-based in terms of existing guidelines on informed decision making (i.e., reporting either benefits or harms but not both; no provision of numbers). When provided with non-evidence-based patient information (*n* = 446), a mean of 33.1% of 235 participants whose initial screening choice contradicted the hypothetical physician's non-evidence-based recommendation adjusted their choice in deference to that recommendation (95% CI: 27.4 to 39.4%), whereas with evidence-based patient information (*n* = 451), only half as many, a mean of 16.0% of 225 (95% CI: 11.8 to 21.4%), modified their choice. Thus, evidence-based patient information makes people less likely to simply follow non-evidence-based recommendations of physicians and supports people in making evidence-based decisions even when not adequately counseled on cancer screening.

## Introduction

Informed medical decision making in medicine, defined as basing one’s decision on the best current medical evidence, requires both informed physicians *and* informed patients. In the traditional view of medicine, physicians hold the knowledge and are mandated to share it with their patients in order to enable them to make an informed choice. In the setting of cancer screening in asymptomatic people, however, where some highly utilized cancer screening tests (e.g., ultrasound for ovarian cancer, mammography, prostate-specific antigen [PSA] screening) have a delicate benefit—harm ratio [1–4], studies showed that a substantial number of physicians do not correctly understand the positive predictive value [5–7] and other statistics [8–10], do not know the scientific evidence for benefits and harms [11, 12], and thus do not possess the preconditions for informed choice.

For instance, in a national study of 412 U.S. primary care physicians, 47% wrongly thought that an increase in screen-detected cancers within the screening group proves that a screening test saves lives, and 76% wrongly believed that a better 5-year survival with screen-detected cancer compared with symptom-detected cancer proves that the test saves lives [9, 13]. However, neither of these statistics delivers that proof [14–16] because of lead-time bias and overdiagnosis bias. For one, screening tends to detect cancer long before it causes symptoms. This simply sets the point of diagnosis earlier, without necessarily affecting the course of life or extending life (lead-time bias), and thus inflates 5-year survival rates. Second, screening inflates the survival and detection rates by including people with non-progressive cancers that by definition do not lead to symptoms or death (overdiagnosis bias). The consequence of these biases is that apparent improvements in survival and detection rates in the screening group as compared to the non-screening group have no bearing on actual improvements in mortality [14].

Physicians have shown similar misconceptions when asked about specific cancer screening tests. For instance, in a cross-cultural survey with 300 U.S. and 300 German urologists, 68% of the U.S. urologists and 75% of the German urologists wrongly believed that the benefit of PSA screening outweighs its harms [17]. Yet a U.S. randomized controlled trial on the effectiveness of prostate cancer screening showed no prostate-cancer-specific mortality reduction at all [1], which has led the U.S. Preventive Services Task Force and other medical organizations to recommend against PSA screening. In Europe, the randomized controlled trial on prostate cancer screening found that the benefit of PSA testing—one man saved from prostate cancer death per 781 men screened for 13 years—is outweighed by its harms by orders of magnitude—27 overtreated men per 781 screened men [18]. And in a convenience sample of German gynecologists who were explicitly asked about the benefits and harms of mammography, not a single gynecologist provided the patient with all the information on the benefit and harms of the screening necessary for making an informed choice [12]. The majority did not provide any numbers on the benefit and harms and the few who did mostly used a technique called *mismatched framing*. Mismatched framing refers to the act of reporting the benefits and harms of a medical intervention in different “currencies,” typically stating the benefits in terms of relative risks (= large numbers) and the harms in absolute risks (= small numbers). For instance, the absolute breast cancer mortality reduction due to mammography screening found in large randomized-controlled trials is from 5 to 4 women out of 1,000 who attended screening for 10 years. This absolute risk reduction of 1 woman saved from breast cancer death per 1,000 can also be expressed as a relative reduction, which is 20% [19]. Whereas the relative risk reduction of 20% looms large to most patients and physicians, the absolute reduction of 1 out of 1,000 does not. Because most people—including pyhsicians—are unaware of the differences between relative and absolute risks, relative risk information results in serious overestimations of the true benefit (e.g., [20, 21]).

Informed decision making is undermined not only by statistical illiteracy but also by the practice of defensive medicine and by conflicts of interest. About 40% of 250 Swiss physicians and 92% of 824 U.S. physicians reported that they order tests for legal, not clinical reasons [22, 23]. And 94% of 1,662 U.S. physicians reported having some type of relationship with the pharmaceutical industry, including payments from the industry in exchange for promoting their products [24].

Given the spread of statistical illiteracy, defensive medicine, and conflicts of interest, one should expect the general public to misperceive the effects of cancer screening. Indeed, in a representative sample of 10,228 men and women in nine European countries, 92% of women and 89% of men either overestimated the reduction of cancer-specific mortality by mammography or prostate cancer screening by a factor of 10 to 200 or did not know the outcome [25]. Of some 1,900 German women regularly attending mammography screening, more than 30% even believed that the screening can prevent breast cancer [26]. In both studies the extent of being misinformed was highest among participants who named their physician as their major source of health information. And in a study focusing on people’s knowledge of the harms (overdiagnosis and overtreatment) of cancer screening, 91% of 317 U.S. citizens age 50 to 69 years said they had not been informed by their physicians about overdiagnosis and overtreatment during their counseling on screening [27].

How can this situation be improved? Specifically, what can be done if we cannot change the incentive structure underlying conflicts of interest and defensive medicine? Although it is often claimed that patients suffer from cognitive deficits that make them predictably irrational when dealing with risks and in need of being “nudged” [28], studies document that evidence-based patient information helps people understand the benefit and harms of medical interventions [26, 29–31] and supports an informed choice [31, 32]. Following the guideline on evidence-based patient information [33, 34] from the German Network for Evidence-based Medicine (DNebM), health information about cancer screening is defined as “evidence-based” if it uses the best available scientific evidence on the benefit *and* harms and presents the information transparently in terms of absolute risk and mortality rate reduction as well as of basic cancer risk specified as age-related prevalence (e.g., out of every 1,000 women aged 50 to 69 years, about 40 people will be diagnosed with the cancer within the next 10 years). In contrast, health information about cancer screening is considered “non-evidence-based” if it displays only partial evidence (e.g., by leaving out information on the harms) and uses nontransparent statistical formats such as relative risks, 5-year survival rates, and annual cancer incidence (e.g., 46,000 women are diagnosed with the cancer every year) without any reference class (e.g., out of 40 million German women). However, given that physicians are the most frequent and trusted source of heath information in the general population in Germany [13], one might not expect the provision of evidence-based patient information alone to have a substantial effect on people’s final choices about cancer screening.

The present study was designed to test empirically whether evidence-based patient information as compared to non-evidence-based patient information about cancer screening supplied to asymptomatic people before counseling can reduce the influence of a subsequent non-evidence-based recommendation by a physician [35]. No previous study appears to have addressed this question.

## Methods

To keep the content of the survey as realistic as possible, the information on the cancer and the cancer screening corresponded to information provided in real patient pamphlets and to medical evidence [19] on breast cancer and mammography screening in Germany. In order to avoid restricting the research question to gender, age, or specific attitudes about mammography screening, the type of cancer and the respective screening remained unspecified in the survey and were called simply “cancer” and “cancer screening.” The content of the survey was reviewed by four physicians (general medicine), piloted with 10 German citizens at target age, and revised according to their feedback. The final survey was approved by the ethics board of the Max Planck Institute for Human Development. It was then implemented in the context of the German Socio-Economic Panel (SOEP)—the longest-running repetition survey panel in the world. Written informed consent to participate in the study was obtained upfront from participants.

The sample frame of the study was the entire German population, divided into 55,000 sample points representative of the population (respondent flow chart, see S1 Respondents). From those 55,000 sample points, 2,504 inhabitants living in private households were preselected by using a standard random-route-walk procedure. Of the preselected sample, 731 participants were excluded from the final sample: 93 participants were used for pilot testing the entire survey and 638 were ineligible for the study because they were less than 18 years old, were unable to speak German, or because the address point turned out not to be a private household. Of the final selected sample of 1,773 eligible participants, 813 declined to participate and 28 provided invalid data in the preface of the survey. Of the remaining 914 eligible participants, 17 were excluded from analysis because of nonresponse to some items, resulting in 897 participants who completed the surveys (response rate: 52%). The final sample is representative of the German population in education and approximates representativeness for gender and age (see Table 1).

**Table 1:** Demographic characteristics of the sample (*N* = 897).

Characteristic	Sample	Population of Germany ≥18 years^b^
	No. (%^a^)	%^a^
Sex		
Female	498 (55)	53
Male	399 (45)	47
Age (years)		
18–29	137 (15)	17
30–39	106 (12)	14
40–49	167 (19)	18
50–59	158 (18)	17
60–69	145 (16)	14
70–79	125 (13)	13
>80	59 (7)	7
Education		
Some high school	331 (37)	37
High school graduate	369 (41)	41
College/university	197 (22)	22

^a^Percentages have been rounded and may not total 100.

^b^Statistisches Bundesamt Deutschland, Wiesbaden. GENESIS-Online-Datenbank, Tabelle 12411–0006, accessed: Dec 16, 2014. https://www-genesis.destatis.de

The sample of 897 citizens in Germany (age 18 and over; Table 1) was surveyed face-to-face by using computer-assisted personal interviewing (CAPI). CAPI is a technique in which the interviewer is physically present to guide the participant through a computerized survey.

The survey consisted of eight cancer screening choice scenarios, which systematically varied on three independent variables: evidence-based versus non-evidence-based patient information about the cancer and the screening test (variable 1); coverage of the screening by health insurance versus coverage by the patients themselves (200 euros) (variable 2); and a non-evidence-based recommendation for or against the screening by a hypothetical physician (variable 3). Participants were randomly allocated to one of eight cancer screening choice scenarios (between-subjects design).

For variable 1, the non-evidence-based patient information about the cancer and the screening test provided in four of the eight scenarios was extracted from actual patient pamphlets for mammography [10, 36–38]. The information consisted of the potentially confusing relative risk reduction of breast cancer death attributable to mammography [21, 39–41], the invalid 5-year-survival rates [14] for describing the benefits of screening, the annual cancer incidence without the respective reference class and did not include information on any harms of screening, such as overdiagnosis. The evidence-based information provided in the other four scenarios was based on the latest Cochrane review on mammography screening [19] and, following the guideline of evidence-based patient information [31, 33, 42], it described both the benefit (cancer-specific death reduction) and harms (including the risk of overdiagnosis) in absolute numbers and mortality rate and also provided the basic cancer risk specified as age-related prevalence [14]. For variable 2, information on financial coverage of the screening was included in the scenarios because some cancer screenings are covered by health plans in Germany (e.g., mammography) while others are not (e.g., prostate cancer screening) and we assumed that payment might moderate the effect of the information format on participants’ personal screening choice. Thus, four of the eight scenarios informed participants that they had to cover the costs (200 euros) of the test themselves, whereas the other four informed them about the coverage by health insurance.

For variable 3, the recommendations of a hypothetical physician in favor of or against screening in both cases did not follow the guidelines for evidence-based patient information. The recommendation provided information either only on the benefits *or* only on the harms of the screening and in both cases was not accompanied by numerical information on the size of either of the outcomes. The wording of the hypothetical physician’s recommendation corresponded to the actual counseling of German gynecologists on mammography screening [9]. In four of the eight scenarios the physician recommended in favor of screening and in the other four against screening.

To determine people’s initial choice, participants were provided with either non-evidence-based or evidence-based patient information about the screening (variable 1) followed by information about financial coverage (covered/200 euros; variable 2) and then asked to make their screening choice. Next, participants were asked to think of their own physician and to imagine that the following recommendation came from him/her. They then learned about the recommendation (variable 3) and were asked to make their final screening choice. At the end, participants were asked to indicate their confidence in that final choice on a 5-point Likert scale. The primary outcome measure of the study was the proportion of participants who changed their initial choice in deference to the physician’s recommendation for their final choice, depending on variable 1 (evidence) and variable 2 (coverage). We did not directly measure trust in a physician’s recommendation but treated the observed effect of that recommendation on participants’ final choice as a surrogate for people’s trust in that advice. The secondary outcome measure was participants’ confidence in their final choice. The exact wording of the survey can be found in the supporting information (S1 Survey). To enhance the reproducibility of science we uploaded our study protocol in protocols.io, where it has been assigned the following identifier (DOI) 10.17504/protocols.io.imscc6e.

All data were stored and analyzed with SPSS 18 (SPSS Inc., Chicago, Ill.). The primary and secondary outcome measures (between-subject differences) as well as the dependence between variable 1 (evidence) and variable 2 (coverage) were analyzed by the nonparametric Pearson chi-square test.

## Results

Of the 897 participants, 451 received evidence-based and 446 non-evidence-based patient information. At the initial decision stage, 53.8% of participants provided with evidence-based information (95% CI: 49.3 to 58.4%, *n* = 451) and 63.3% of those provided with non-evidence-based information (95% CI: 58.7 to 67.6%, *n* = 446) decided in favor of screening, resulting in a difference of 9.6 percentage points. Information on the coverage of the cost of screening had a similar influence on participants' initial screening choice: 63.8% (95% CI: 59.2 to 68.1%, *n* = 447) decided in favor of screening when covered and 53.3% (95% CI: 48.7 to 57.9%, *n* = 450) initially chose screening when they had to pay 200 euros.

**Fig 1:**
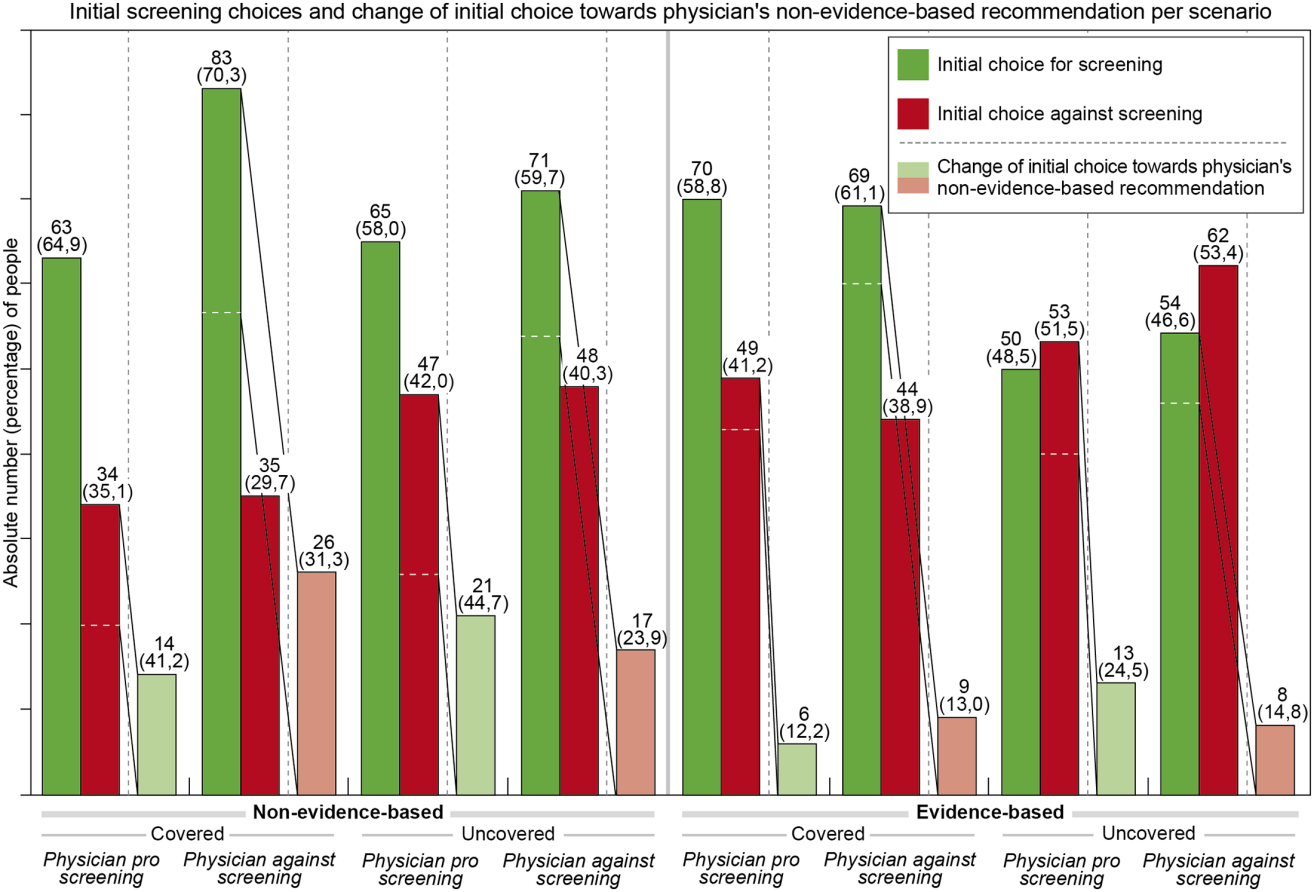
Evidence-based patient information given before counseling reduces the influence of non-evidence-based counseling by physicians.

After participants who were presented with non-evidence-based patient information received a non-evidence-based recommendation by a hypothetical physician, 23.9% to 44.7% of the 235 participants (*M* = 33.1%, 95% CI: 27.4 to 39.4%) whose initial choice diverged from that advice adjusted their choice in deference to the respective recommendation (see Fig 1). Among those who had received evidence-based patient information, only 12.2% to 24.5% of the 225 participants (*M* = 16.0%, 95% CI: 11.8 to 21.4%) whose initial choice diverged from the physician’s non-evidence-based advice adjusted their initial choice accordingly (see Fig 1). Evidence-based information thus more than halved the impact of a hypothetical physician’s non-evidence-based recommendation on people’s final screening choice (Pearson’s χ^2^ = 15.47, *df* = 1, *p* < .001) (see Fig 1). Unlike the variable of (non-)evidence-based information, coverage of screening had no discernable effect on the impact of a physician’s recommendation on respondents’ screening choice (Pearson’s χ^2^ = 0.14, *df* = 1, *p* = .71): With coverage, a mean of 23.4% of 235 participants (95% CI: 18.5 to 29.2%) adjusted their initial decision to match the physician’s recommendation compared to a mean of 26.2% of 225 participants (95% CI: 20.9 to 32.3%) without coverage (see Fig 1).

Testing for dependence between the variables 1 (evidence) and 2 (coverage) showed that the two variables were independent of one another (Pearson’s χ^2^ = 0.56, *p* = .45, φ = .09).

The proportion of participants who were confident to very confident in their choice to decide against a physician’s recommendation did not differ between participants who received the non-evidence-based information (*M* = 76.3%, 95% CI: 68 to 82%) and those who received evidence-based information (*M* = 69.3%, 95% CI: 62 to 75%) nor between participants who received a hypothetical physician’s recommendation favoring screening (*M* = 65.9%, 95% CI: 57 to 75%) and those who received a recommendation against screening (*M* = 74.6%, 95% CI: 68 to 80%). Overall, however, more participants felt confident to very confident when their final choice was in accordance with the hypothetical physician’s recommendation (*M* = 76.5%, 95% CI: 73 to 79%) than when it was not (*M* = 62.4%, 95% CI: 55 to 70%).

## Discussion

For a national sample of adults in Germany we showed that the provision of evidence-based patient information before counseling reduced the influence of a physician’s non-evidence-based screening recommendation on people’s final screening choice by more than half. The results indicate that providing people with evidence-based patient information by itself supports them in making evidence-based decisions in situations where physicians’ screening recommendations do not follow the guidelines of evidence-based patient counseling. Crucially, such support for more evidence-based patient decisions can be gained without the help of statistically literate physicians or changes in the incentive structure underlying conflicts of interest and defensive medicine. Evidence-based decision making is especially important in cancer screening of asymptomatic people because there is no medical urgency for intervention or treatment and therefore choices are made in a preference-sensitive decision setting.

The strength of the study lies in it being, to the best of our knowledge, the first to investigate the effect of evidence-based versus non-evidence-based patient information on people's decisions whether to follow non-evidence-based screening recommendations. The results are based on a study of a nationwide sample of the German general public, using the actual phrasing of German cancer screening pamphlets together with actual physicians’ recommendations documented in a previous study.

The limitations of the study are that screening intentions as opposed to actual screening decisions were measured, and that the hypothetical scenario and physician do not fully capture the intensity of a real physician—patient relationship. It would be nearly impossible, however, to conduct the present study in a true clinical situation because physicians would undoubtedly feel uncomfortable about deliberately providing non-evidence-based information and awareness of the intentions behind the study could influence their counseling. Thus, using the scenario technique appeared to be the most realistic method to learn about the effect of evidence-based information on following non-evidence-based advice. It might be further argued that in their cancer screening counseling of asymptomatic patients, physicians take more into consideration than just medical evidence, such as the specific cancer and the patient's clinical history. However, recent U.S. screening uptake rates—according to the Behavioral Risk Factor Surveillance System Survey 2015—do not support the assumption that current clinical recommendation practices are tailored to specific cancers or patients’ clinical history. To further ensure ecological validity of the study the non-evidence-based format was based on real examples of German patient pamphlets. Due to that fact, the non-evidence-based format and the evidence-based format, which was based on standards for best-practice patient information, may have differed in their persuasiveness of tone as well as in their degree of complexity.

The intention of our study should not be misconstrued as a call against trusting physicians’ recommendations. Trust in physicians is an important prerequisite for an effective health care. Yet, the study takes a stand against unconditional trust in non-evidence-based recommendations that hinder informed decision making. Studies have suggested that non-evidence-based recommendations in the context of screening are more the rule than the exception for various reasons. The most prominent is that a considerable number of physicians themselves do not correctly understand their own medical statistics [8, 9, 12]. Past studies found that physicians are susceptible to framing effects created by using relative as opposed to absolute risk reduction formats [20, 41, 43, 44], have difficulty calculating the positive predictive value [5, 6, 45, 46], and are easily misled by 5-year survival rates in the context of screening [8, 9]. Misunderstanding of statistics matters, since it influences how physicians discuss screening with their patients or how they teach trainees. Physicians who are not able to draw correct conclusions from existing medical evidence are unlikely to fully inform their patients about the benefits and harms of screening tests [5, 12, 27], which in turn renders the patient unlikely to make an informed decision about cancer screening. Conflicts of interest and the practice of defensive decision making [23, 47] may further lead to recommending cancer screening tests sometimes even despite the fact that major medical organizations do not recommend these. Bearing these limitations in mind, the provision of evidence-based patient information to people can make for a considerable change towards informed decision making.

## Conclusions

The practical consequence of the present study is to substantially increase the transparency of information based on best medical evidence in screening pamphlets and websites in order to support patients in making evidence-based decisions even if their physicians have conflicts of interest, practice defensive medicine, or lack statistical literacy. At present, health organizations and charities tend to oversell screening [48] and screening pamphlets tend to use nontransparent information [37, 49, 50]. The results should also inspire medical educators to consider adjusting their curricula to ensure that every medical student receives adequate training in correctly understanding and transparently communicating medical evidence about cancer screening. The findings further strongly support initiatives of medical journal editors on transparent and evidence-based reporting of study results [51] as well as of medical scientists on the provision of transparent and evidence-based patient information tools such as fact boxes [52] as first promising steps toward attaining the ideal of informed decision making. Evidence and transparency in medicine is the necessary condition for a trustful relationship between physicians and patients as well as for informed decision making. If physicians wish to rightfully remain patients' most trusted and reliable source of information, the medical profession needs to not just encourage but also enforce evidence and transparency on all levels, from education to reporting.

## Declarations

**Ethical approval**: The study was approved by the institutional ethics board of the Max Planck Institute for Human Development, Berlin (Germany).

**Consent to participate**: Written informed consent to participate in the study was obtained upfront from participants.

**Consent to publish**: We have obtained consent from participants to publish and report their individual data as aggregated data. We have not obtained consent to publish individual data, because no individual data are presented within the manuscript.

## Supporting information

S1 Respondents Respondents flow chart. (PDF)

S1 Survey Exact wording of the study survey. (PDF)
