# Supplementary figures and images for: Can facts trump unconditional trust? Evidence-based information halves the influence of physicians’ non-evidence-based cancer screening recommendations
Source: PLoS One. 2017 Aug 23;12(8):e0183024. doi: 10.1371/journal.pone.0183024 (PMC5568103; doi:10.1371/journal.pone.0183024)

## S1\_Respondents

### Respondent flow chart

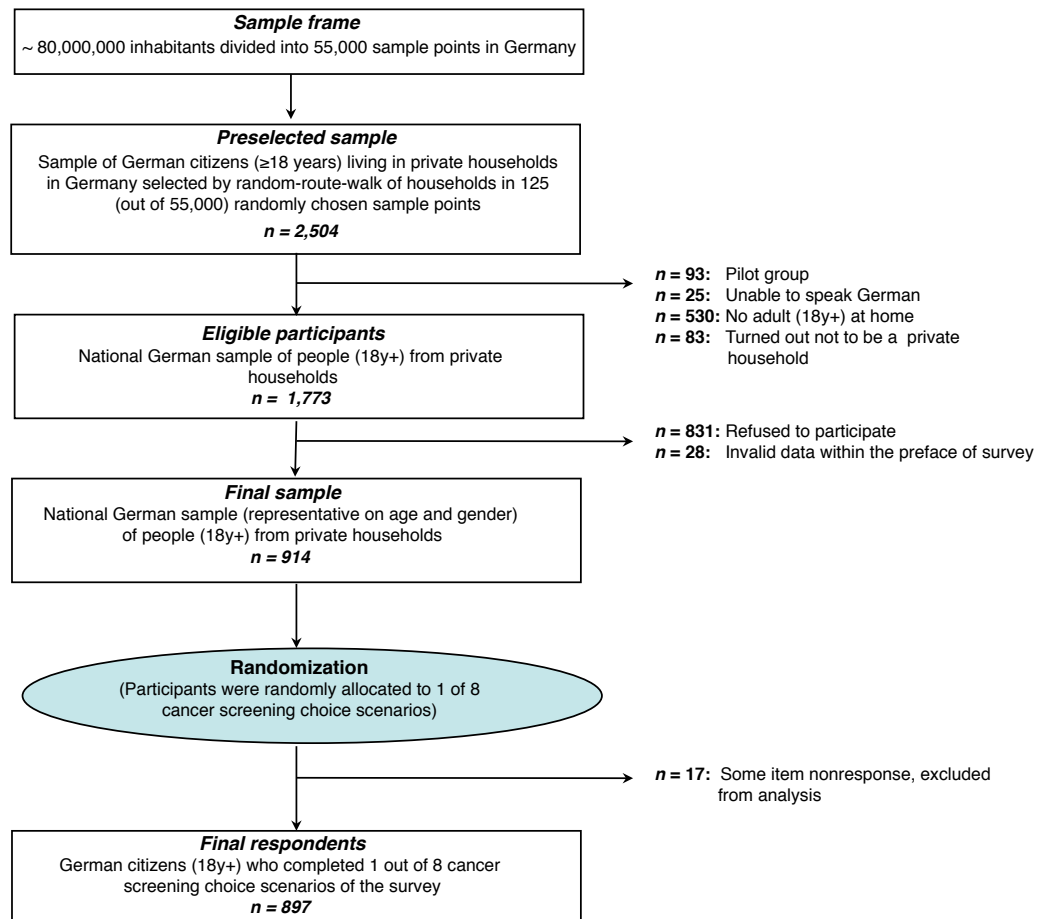

Supplement: S1 Respondents — (PDF) [file pone.0183024.s001.pdf]
