# Supplementary material for: Can facts trump unconditional trust? Evidence-based information halves the influence of physicians’ non-evidence-based cancer screening recommendations
Source: PLoS One. 2017 Aug 23;12(8):e0183024. doi: 10.1371/journal.pone.0183024 (PMC5568103; doi:10.1371/journal.pone.0183024)
Supplement: S1 Survey — (PDF) [file pone.0183024.s002.pdf]

S1_Survey

Survey: Variables, construction of scenarios, and example of a scenario

(Please note, the survey is a translation of a German survey protocol)

Featured variables per scenario:

Scenario 1: Variable 1: Non-evidence-based patient information, Variable 2: Coverage, Variable 3: Physician recommends non-evidence-based for screening

Scenario 2: Variable 1: Non-evidence-based patient information, Variable 2: Coverage, Variable 3: Physician recommends non-evidence-based against screening

Scenario 3: Variable 1: Non-evidence-based patient information, Variable 2: No coverage, Variable 3: Physician recommends non-evidence-based for screening

Scenario 4: Variable 1: Non-evidence-based patient information, Variable 2: No coverage, Variable 3: Physician recommends non-evidence-based against screening

Scenario 5: Variable 1: Evidence-based patient information, Variable 2: Coverage, Variable 3: Physician recommends non-evidence-based for screening

Scenario 6: Variable 1: Evidence-based patient information, Variable 2: Coverage, Variable 3: Physician recommends non-evidence-based against screening

Scenario 7: Variable 1: Evidence-based patient information, Variable 2: No coverage, Variable 3: Physician recommends non-evidence-based for screening

Scenario 8: Variable 1: Evidence-based patient information, Variable 2: No coverage, Variable 3: Physician recommends non-evidence-based against screening

Content of each variable:

Variable 1: Non-evidence-based versus evidence-based patient information:

Non-evidence-based:

Imagine that you have to make a decision whether to participate in a cancer screening test. The following is what you know:

- Every year 46.000 people are diagnosed with the cancer that the screening is for.
- By participating in the cancer screening you can reduce your risk of dying of that cancer by up to 30%.
- Also, people whose cancer is caught early by the screening have a 98% 5-year survival rate.
- The screening procedure uses X-ray technology. However, the devices are of highest modern standard, which keeps the radiation exposure very low.

Evidence-based:

Imagine that you have to make a decision whether to participate in a cancer screening test. The following is what you know:

- Out of every 1,000 people aged 50 to 69 years, about 40 people will be diagnosed with the cancer within the next 10 years.
- Without screening about 4 people will die of the cancer within the next 10 years. By participating regularly in the screening over a time frame of 10 years, one less person will die of this cancer.

- The screening can result in a wrong diagnosis. Of 1,000 people attending screening, about 100 will receive a false alarm, which is corrected for most by further testing. However, five people will mistakenly receive a final diagnosis of cancer and subsequently receive cancer treatment (e.g., surgery, radiation, chemotherapy) although they do not have the cancer.
- The screening procedure uses X-ray technology. Up to now, it is unclear if and how many additional cancer incidences are provoked by the X-ray procedure. Rough estimates assume 1 additional cancer case per 10,000 people regularly attending that screening.

Variable 2: Coverage versus no coverage

Coverage:

- The screening is recommended biannually and covered by your health insurance company.

No coverage

- The screening is recommended biannually. The screening costs 200 euros and has to be covered by yourself.

Given this information, would you consider participating in the cancer screening?

Yes □

No □

Variable 3: Physician's recommendation non-evidence-based for versus against screening

Physician's recommendation non-evidence-based for screening

Now think as intensively as possible of your family doctor.

- Imagine that your doctor firmly recommends participating in screening as it would increase your chances of surviving the cancer and improve therapeutic control of the cancer.

Physician's recommendation non-evidence-based against screening

Now think as intensively as possible of your family doctor.

- Imagine that your doctor tells you that is unclear whether the benefit outweighs the harms such as overdiagnosis and overtreatment of the screening and recommends against it.

How would you eventually decide about the screening?

Yes, I would participate in that screening. □

No, I would not participate in that screening. □

On a scale of 1 (= not at all certain) to 7 (= extremely certain), how confident do you feel with your final decision?

1<br>Not at all certain	2	3	4	5	6	7<br>Extremely certain

Example of scenario 1:

Imagine that you have to make a decision whether to participate in a cancer screening test. The following is what you know:

- Every year 46.000 people are diagnosed with the cancer that the screening is for.
- By participating in the cancer screening you can reduce your risk of dying of that cancer by up to 30%.
- Also, people whose cancer is caught early by the screening have a 98% 5-year survival rate.
- The screening procedure uses X-ray technology. However, the devices are of highest modern standard, which keeps the radiation exposure very low.

- The screening is recommended biannually and covered by your health insurance company.

Given this information, would you consider participating in the cancer screening?

Yes ◻

No ◻

Now think as intensively as possible of your family doctor.

Imagine that your doctor firmly recommends participating in screening as it would increase your chances of surviving the cancer and improve therapeutic control of the cancer.

How would you eventually decide about the screening?

Yes, I would participate in that screening. ◻

No, I would not participate in that screening. ◻

On a scale of 1 (= not at all certain) to 7 (= extremely certain), how confident do you feel with your final decision?

1<br>Not at all certain	2	3	4	5	6	7<br>Extremely certain
